# Exploring the implemented guidelines for dyslipidemia treatment and care among nurses and physicians: A qualitative study in Jordan

**DOI:** 10.1371/journal.pone.0319126

**Published:** 2025-08-07

**Authors:** Rana bani Salameh, Ahmed Al-Smadi, Omar Gammoh, Abedalmajeed Shajrawi, Ala Ashour, Omar Alrfooh, Donna Fitzsimons, Taher Hatahet, Ammena Yahia Binsaleh, Sireen Abdul Rahim Shilbayeh

**Affiliations:** 1 Department of Adult Nursing Care, Faculty of Nursing, Al al-Bayt University, Al-Mafraq, Jordan; 2 Faculty of Nursing, Zarqa University, Zarqa, Jordan; 3 Nursing department, Fakeeh college for medical sciences, Jeddah, Saudi Arabia; 4 Department of Clinical Pharmacy and Pharmacy Practice, Faculty of Pharmacy, Irbid, Yarmouk University, Jordan; 5 Faculty of Health Sciences, Sharjah Campus, Higher Colleges of Technology, United Arab Emirates; 6 Department of Allied Medical Sciences, Faculty of Applied Medical Sciences, Jordan University of Science and Technology, Irbid, Jordan; 7 School of Nursing and Midwifery, Queen’s University Belfast, Belfast, United Kingdom; 8 School of Pharmacy, Queen’s University Belfast, Belfast, United Kingdom; 9 Department of Pharmacy Practice, College of Pharmacy, Princess Nourah bint Abdulrahman University, Riyadh, Saudi Arabia; University of Diyala College of Medicine, IRAQ

## Abstract

**Background:**

Dyslipidemia is a major risk factor for ischemic heart disease worldwide. The most recent guidelines from the American College of Cardiology/American Heart Association (ACC/AHA) and the European Society of Cardiology/European Atherosclerosis Society (ESC/EAS) aim to control cholesterol levels and reduce cardiovascular risk. In Jordan, where no national guidelines exist, international guidelines are commonly adopted. However, limited research has examined their implementation in clinical practice. This study aimed to explore the implementation of updated ACC/AHA and ESC/EAS guidelines for the treatment and care of dyslipidemia among nurses and physicians in Jordan.

**Methods:**

Semi-structured focus group interviews with open-ended questions were conducted to gain a deeper understanding of current practices and guideline implementation among nurses and physicians.

**Results:**

The focus group discussions revealed that healthcare professionals attempt to follow specific guidelines and achieve optimal treatment goals. However, several barriers, including a heavy workload, limited resources, and a lack of institutional support, hinder effective implementation. Participants also provided several suggestions to improve guideline implementation and expressed the need for national, context-specific dyslipidemia management guidelines in Jordan.

**Conclusions:**

The study found limited implementation of dyslipidemia management guidelines among Jordanian nurses and physicians in clinical practice. Findings highlight the need for national guidance and system-level support to facilitate better implementation.

## Introduction

Dyslipidemia is defined as “elevated total cholesterol (TC), low-density lipoprotein cholesterol (LDL-C), or triglycerides (TG); low high-density lipoprotein cholesterol (HDL-C); or a combination of these abnormalities” [1]. In 2019, WHO considered dyslipidemia a major risk factor for two causes of death in the world: ischemic heart disease (IHD) and stroke [2]. In 2017, the WHO estimated the worldwide deaths from IHD and ischemic stroke at 3.9 million (3.7–4.2 million) for one type of dyslipidemia (high non-HDL cholesterol) [3]. Each year, 4 million people in the United States and 1.5 million in the European Union die from cardiovascular disease (CVD), according to the European Society of Cardiology (ESC) [4].

In 2018, CVDs accounted for 37% of all deaths in Jordan [5]. A Jordanian cohort study identified hypertension, diabetes, hyperlipidemia, and coronary artery disease as the leading risk factors for ischemic stroke [6]. A national prevalence study conducted in 2017 on the lipid profile in Jordan reported that 44.3% had high TC, 41.9% had high TG, 75.9% had high LDL-C, and 59.5% had low HDL-C [7].

Physicians and nurses play a major role in the treatment and management of dyslipidemia [8]. Few studies have explored the implementation of dyslipidemia management guidelines among nurses and physicians in Jordan. Halawani et al. (2019) highlighted the importance of physicians’ knowledge of the latest guidelines in improving patient care quality, which leads to better lipid profile outcomes [9]. Another study in Jordan reported a low level of knowledge regarding the American College of Cardiology/American Heart Association (ACC/AHA) guidelines among Jordanian physicians [10]. Furthermore, a separate study conducted in Jordan showed awareness rates of dyslipidemia (9.3%), treatment (50.3%), and control (25.4%) among adults [11]. The most recent ACC/AHA and European Society of Cardiology/European Atherosclerosis Society (ESC/EAS) guidelines for dyslipidemia management aim to control blood cholesterol and reduce cardiovascular risk [12,13].

To the best of our knowledge, no national guidelines for dyslipidemia management have been published in Jordan to date, posing a significant challenge for healthcare professionals. Consequently, healthcare providers primarily rely on international guidelines, particularly those from the ACC/AHA and ESC/EAS. Due to the lack of prior research on this topic, this study was conducted to explore the implementation of the updated ACC/AHA and ESC/EAS guidelines among nurses and physicians, assess the level of treatment control among patients with dyslipidemia, and identify barriers and suggestions for guideline implementation in Jordan.

## Method

A qualitative descriptive design was used to explore the implementation of dyslipidemia treatment guidelines among nurses and physicians in Jordanian hospitals. This design was chosen because it is appropriate for gaining a detailed understanding of participants’ real-life experiences, perspectives, and the contextual challenges involved in implementing clinical guidelines. The study involved three semi-structured focus group interviews. Participants were recruited from two public Jordanian hospitals and one teaching hospital: King Abdullah University Hospital, Al Basheer Governmental Hospital, and Al Mafraq Governmental Hospital. These hospitals are located in two geographical regions in northern and central Jordan.

### Sample

Three focus groups were conducted, each consisting of at least one cardiologist, one internal medicine physician, and four nurses from public Jordanian hospitals. A purposive sampling method was used to recruit a total of 20 healthcare professionals (12 nurses and 8 physicians) from four cardiac units within each hospital: the critical care unit, intensive care unit (ICU), intermediate cardiac care unit, and the medical and surgical cardiac ward. Each focus group included participants from a single hospital, representing diverse roles and experiences within cardiology and cardiac surgery departments.

Inclusion criteria required that participants be registered nurses or physicians with at least one year of continuous clinical experience in their current cardiac unit. They also had to be actively involved in patient care and familiar with the implementation of clinical guidelines. The researcher conducted an introductory meeting to invite eligible individuals to participate in the study. Participants were selected using a purposive sampling method [14].

Three hospitals were selected to ensure diverse representation of healthcare settings. One central hospital and one teaching hospital were included to provide in-depth data from high-resource environments, while an additional hospital from a smaller, regional setting was selected for comparison.

The sample size was primarily guided by the principle of data saturation, as recommended in qualitative research. Data collection concluded when no new themes or insights emerged from the interviews, indicating that the data were sufficient to address the study objectives and capture the depth of participants’ perspectives. Practical constraints, including time and available resources, were also considered. While the sample size was limited, it was adequate for the exploratory aims of this study and provided valuable insights into the influence of hospital resources on clinical practice. The sample size aligns with recommendations from Creswell and Poth, and Polit and Beck, who emphasize data saturation and the information needs of the research as key determinants for qualitative sample sizes [15].

### Ethical consideration

Ethical approval was obtained from the Committee of Scientific Research and Ethics at the Faculty of Nursing, Al al-Bayt University, Jordan (Reference Number: 07/2021/2022; Date of Approval: 23 May 2022), as well as from the institutional review boards of each hospital included in this study. All participants were volunteers and remained anonymous. Written informed consent was obtained from all participants before data collection. Participant confidentiality was strictly maintained throughout the study.

### Tools for the data collection procedure

Focus group semi-structured interviews were conducted with physicians and nurses using an interview guide developed from the literature (File 1). The guide included open-ended questions focused on current practices and guideline implementation for dyslipidemia treatment and care in Jordan, as well as perceived barriers and suggestions for improving implementation. Mixed focus groups were conducted in each hospital, with both physicians and nurses participating. Each group consisted of five to ten participants. A preliminary meeting was held to form the groups, during which participants were informed about the purpose of the focus groups and the study objectives. Informed consent was obtained from all participants (File 2). All interviews were audio-recorded and transcribed verbatim in Arabic, capturing participants’ exact words (File 3). The transcripts were then carefully translated into English to ensure the accuracy and preservation of the original meaning.

### Data collection procedure

The researcher approached 20 healthcare professionals during their clinical work who met the inclusion criteria and invited them to participate in semi-structured focus group interviews after fully explaining the study. Consent forms were provided, and participants were asked to suggest a convenient date and time for the interview. After obtaining informed consent and demographic information, the semi-structured focus group interviews were conducted in Arabic in a quiet and private setting within the hospital. All interviews were audio-recorded. At the beginning of each session, the researcher introduced the study, explained the specific objectives of the interview, and ensured that participants were comfortable. The recording equipment was set up, and a brief informal conversation was initiated to build rapport, followed by the open-ended questions guided by the interview schedule. Each interview lasted approximately 20 minutes, which was acceptable to participants considering their clinical responsibilities and was sufficient to achieve data saturation. All interviews were conducted by the first author. Data collection took place between June 1 and August 1, 2022.

### Thematic analysis

The primary aim of the analysis was to explore in depth the implemented practices, perceived barriers, and suggestions for guideline implementation among nurses and physicians. Data were analyzed using thematic analysis, following Braun and Clarke’s approach [16]. This method involves six interrelated but distinct phases: (1) data immersion, (2) generating initial codes, (3) searching for themes, (4) reviewing themes, (5) defining and naming themes, and (6) producing the report.

All interviews were audio-recorded and later transcribed verbatim in Arabic, ensuring that participants’ words were captured exactly as spoken. The transcriptions were then carefully translated into English to maintain accuracy and preserve the original meaning. The transcripts were anonymized by removing names and personally identifying information, and each interview was assigned a unique identifier or code.

In the data immersion phase, the researcher read and re-read the transcripts and re-listened to the audio recordings to become deeply familiar with the data. During the initial coding phase, transcripts were reviewed line by line, and labels were assigned to significant statements or ideas. Similar or related codes were then grouped into broader categories representing shared concepts. These categories were systematically applied across the dataset. When no new codes emerged, theoretical saturation was considered to be reached [17]. Each code was documented using a number or abbreviation directly on the transcripts (File 4).

The next phase involved generating themes by identifying patterns and commonalities across codes. Similar codes were clustered into broader themes that reflected key findings in the data. In the reviewing stage, themes were refined by comparing them against the original transcripts to ensure they were grounded in the participants’ narratives. Finally, each theme was clearly defined and described to reflect its content and relevance to the research aims.

### Measures for ensuring trustworthiness

To enhance the rigor and quality of the findings, the researcher evaluated the trustworthiness of the qualitative data using established criteria [18].

### Credibility

Credibility was ensured through member checking. Interview transcripts were returned to all participants, who were invited to review the content for accuracy. Participants were asked to verify or clarify their responses to ensure that their views were accurately represented.

### Transferability

Transferability was addressed by conducting the study in three different healthcare settings that represent the public healthcare sector in Jordan. Participants were recruited from diverse cardiac units and included both nurses and physicians, which supports the applicability of the findings to similar contexts.

### Dependability

Dependability was strengthened by collecting data in appropriate settings, at suitable times, and from relevant participants. An audit trail was maintained to document each stage of the research process, including data collection, analysis, and decision-making.

### Confirmability

Confirmability was established by clearly describing the study methods and procedures, allowing readers to trace how the data were collected, processed, analyzed, and interpreted. The entire research process and analytic steps were reviewed and discussed with the study team, supervisors, and a qualified clinical expert to reduce potential researcher bias.

## Results

As described in the methodology, focus group interviews were audio-recorded after forming the groups and obtaining written consent from participants. An interview guide with open-ended questions was used (File 1). The analysis resulted in three main themes (Table 1). Each theme is discussed in detail in this section and supported with direct participant quotations.

**Table 1:** Themes and sub-themes emerging from the interview data.

Themes	Sub-themes	Description/ Expanded Points
1. Perceived different barriers that affect the implementation of dyslipidemia management guidelines in Jordan	A. Patient-related barriers	- Non-compliance with medications and lifestyle advice - Lack of follow-up and lipid profile monitoring - Psychological resistance and cultural misconceptions
	B. Healthcare professional-related barriers	- Lack of training or updates on guidelines - Poor communication with patients - Limited collaboration between physicians and nurses
	C. Healthcare system-related barriers	- Shortage of medical staff - Limited availability or high cost of medications - Lack of institutional support - Heavy workload and time constraints
2. Perceived different suggestions and recommendations that affect implementation	A. Structural recommendations	- Establishment of specialized dyslipidemia clinics - Development of national, Jordan-specific guidelines
	B. Awareness and screening	- Public awareness campaigns - Offering free lipid profile screening to the public
	C. Clinical practice improvements	- Regular training programs for healthcare professionals - Organized follow-up systems for patient monitoring
3. Perceived limited implementation of dyslipidemia management guidelines in Jordan	A. Implemented practice strategies	- Relying on clinical experience over standardized protocols - Use of general practices without reference to guidelines
	B. Inconsistency in following guidelines	- Limited awareness of international guidelines - Absence of formal institutional policies - Partial or selective application of guideline components

*Note: Source original.*

### 1) Perceived different barriers that affect the implementation of dyslipidemia management guidelines in Jordan

The theme included the following sub-themes: patient-related barriers, health care professional barriers, and health care system-related barriers.

#### A: Patient-related barriers: sub-theme.

Sixty percent of participants (n = 12) reported that patients often lacked awareness and understanding of the chronic nature of dyslipidemia, which led to poor medication adherence and limited engagement with recommended lifestyle modifications. Many participants noted that patients did not perceive dyslipidemia as a serious or long-term condition, especially in the absence of symptoms. This perception was attributed to both cultural beliefs and insufficient health education.

*“One of the most significant barriers affecting the implementation of dyslipidemia management guidelines in Jordan is the lack of compliance. Patients often believe that medication is only needed for one month and are unaware that they must continue treatment indefinitely. Many do not undergo lipid profile tests, eat healthy foods, or follow recommended diets and lifestyles.”* (Cardiologist, 9)

Forty percent of participants (n = 8) stated that even when they advise their patients, many do not adhere to the recommendations, particularly regarding lifestyle changes.

*“Even if I advise my patients, a large number of them do not follow it, particularly when it relates to lifestyle changes.” (Registered nurse,11)*

*“They do not follow up, even after we explain their condition. They say they feel better and stop everything.”* (Registered nurse, 6)

In addition to poor adherence, logistical and behavioral factors were also reported. Twenty-five percent of participants (n = 5) noted that patients often avoid long waiting times in public hospitals and instead purchase medications directly from private pharmacies without adequate follow-up or clinical monitoring.

*“Patients often dislike waiting for long hours for their appointments and prefer to purchase their medications from private pharmacies without attending follow-up visits.”* (Registered nurse, 11)

This pattern reveals a common misalignment between the goals of evidence-based guidelines and patients’ lived experiences and expectations. Thirty percent of participants (n = 6) described psychological resistance, fatalistic attitudes, and a low perceived need for ongoing care as significant obstacles to long-term management.

#### B: Health Care Professional Related Barriers: Sub-Theme.

Fifty percent of participants (n = 10) identified gaps in their preparedness to implement guidelines, including limited training opportunities, outdated knowledge, and weak interdisciplinary communication.

*“We have a lack of knowledge about dyslipidemia management guidelines. There is no training course for nurses and physicians.”* (Registered nurse,3)

Thirty percent of participants (n = 6) reported weak communication among healthcare professionals and between providers and patients.

*“There is no good communication between patients and physicians and nurses.”* (Internal medicine physician, 1)

Twenty-five percent of participants (n = 5) expressed a sense of exclusion, particularly among nurses who felt their role in lipid management was marginal.

*“Nurses aren’t usually involved in lipid management decisions, so we don’t feel part of the treatment process.”* (Registered nurse, 5)

These findings suggest that, beyond structural limitations, professional identity and role clarity issues hinder effective team-based implementation. The absence of training not only limits knowledge but also affects confidence and initiative.

#### C: Health Care System Related Barriers: Sub-Theme.

Fifty percent of participants (n = 10) discussed system-level barriers as major obstacles to implementing dyslipidemia management guidelines. Participants identified chronic understaffing, lack of institutional support, and inconsistent availability of medications as critical factors that undermine the feasibility of guideline adherence in clinical practice.

*“The main barrier that faces us is the shortage of physicians and nurses.”* (Internal Medicine 1)

*“Some of the medications are not available or are too expensive. Other medications are sometimes available and sometimes not. That prevents us from implementing the guidelines.”* (Registered nurse,4)

Thirty percent of participants (n = 6) highlighted time constraints due to overwhelming patient loads, which further compounded the problem and limited the capacity of providers to implement guideline-based care.

“*We don’t have enough time to implement dyslipidemia management guidelines in our hospitals or address patient culture.”* (Registered nurse, 3)

Twenty-five percent of participants (n = 5) also noted the absence of a structured follow-up system, which hinders continuity of care and limits long-term management.

*“There’s no structured system to follow up with patients. If they miss appointments, nobody tracks them.”* (Cardiologist, 9)

These insights emphasize how structural deficiencies at the health system level directly impact the ability of providers to follow through with evidence-based practices. The lack of institutional mechanisms for patient tracking or continuity of care was seen as a major missed opportunity.

### 2) Perceived different suggestions and recommendations that affect the implementation of dyslipidemia management guidelines in Jordan

Participants provided a range of practical suggestions aimed at improving the implementation of dyslipidemia guidelines. These were categorized into three sub-themes: structural solutions, awareness and screening, and improvements in clinical practice. The responses reflected an awareness of both system-level needs and population-level gaps.

Thirty-five percent of participants (n = 7) emphasized the need for structural solutions, particularly the establishment of specialized dyslipidemia clinics and the development of locally relevant national guidelines. These suggestions stemmed from perceptions that current hospital systems are too overwhelmed to properly manage chronic lipid disorders.

*“One of the solutions is to make a clinical center to treat hyperlipidemia in Jordan like KHCC... these patients should be transferred to the dyslipidemia center.”* (Cardiologist, 9)

*“We need our guidelines in Jordan, adapted to our patients, not just to apply European or American guidelines.”* (Internal Medicine, 1)

This indicates a desire for context-sensitive structures that support implementation beyond individual efforts.

Thirty percent of participants (n = 6) highlighted the importance of public awareness and early screening. They noted that public knowledge about dyslipidemia is generally low and suggested mass education campaigns and free screening programs to enhance early detection and improve treatment adherence.

*“Start public awareness campaigns on TV and social media to educate people about lipids and heart health.”* (Registered nurse, 6)

Twenty-five percent of participants (n = 5) focused on improvements in clinical practice. They called for more organized follow-up systems and routine training programs for healthcare workers to enhance guideline implementation.

*“There must be regular training sessions for physicians and nurses to refresh their knowledge.”* (Registered nurse, 3)

Overall, these suggestions reflect an understanding that successful implementation depends not only on clinical expertise but also on system-wide support, community engagement, and continuous education.

### 3) Perceived limited implementation of dyslipidemia management guidelines in Jordan

Sixty-five percent of participants (n = 13) acknowledged that the formal implementation of dyslipidemia management guidelines in their institutions was weak or absent. Many reported relying on personal clinical experience rather than structured protocols. This theme reflects a significant gap between knowledge and practice, shaped by systemic barriers and a high degree of professional autonomy.

*“We don’t follow any guidelines for dyslipidemia management in our hospital”.* (Registered nurse, 2)

*“I just follow my experience.”* (Internal Medicine, 1)

Thirty percent of participants (n = 6) indicated that the limited application of guidelines was due to the absence of institutional policies, lack of accountability, and insufficient training.

“*We have a guideline, but we don’t apply it because we don’t have a correct follow-up”*. (Cardiologist,9)

Additionally, twenty-five percent of participants (n = 5), mostly nurses, reported being excluded from decision-making processes, which further limited their involvement in the application of dyslipidemia management guidelines.

*“Nurses are not involved in applying the guideline... we don’t really know if it’s guideline-based.”* (Registered nurse, 4)

This theme illustrates how systemic, cultural, and professional factors intersect to hinder consistent guideline adoption in clinical settings.

## Discussion

The qualitative findings revealed that the majority of participants did not use any recommendations in their clinical practices, while some attempted to follow easier and more up-to-date rules. However, all participants stated that they manage patients with dyslipidemia based on their experience, and some nurses stated that the guidelines constitute medical management rather than nursing management. Previous findings suggested that the dyslipidemia care guidelines were not implemented and followed by all participants, and they relied on their clinical experience to handle patients with dyslipidemia.

This gap between knowledge and practice underscores a broader challenge within healthcare systems where clinical guidelines are not adequately translated into day-to-day workflows. While the presence of guidelines may suggest theoretical awareness, the lack of practical integration suggests insufficient institutional support, absence of accountability, and poor implementation infrastructure. These findings are consistent with research conducted in Saudi Arabia and Jordan, which revealed a low level of awareness and understanding of the 2013 ACC/AHA guidelines among physicians and clinical pharmacists [10,19]. In both contexts, knowledge gaps translated directly into weak adherence, highlighting a common pattern in the region.

Furthermore, the reported dependence on individual experience rather than standardized approaches may reflect a fragmented care culture where interdisciplinary collaboration is minimal and continuing education opportunities are scarce. This dynamic reinforces inequities in care delivery and increases the risk of inconsistency in managing dyslipidemia across settings.

The current study revealed several systemic and practical challenges that contribute to the low adoption of dyslipidemia guidelines in clinical practice. Participants described barriers such as limited resources, time constraints, insufficient training opportunities, and a lack of institutional support. These challenges are not only logistical but also reflect deeper organizational and structural issues that hinder the integration of evidence-based practices. Similar difficulties have been reported in previous studies, highlighting the complexity of applying clinical guidelines in everyday healthcare [20–23].

In addition to these systemic barriers, the study also highlighted the significant role of workplace culture and individual attitudes in influencing guideline adherence. Many healthcare providers tend to rely on their personal clinical experience and question the practical relevance of guidelines in their specific settings. This skepticism may undermine efforts to implement guidelines even when institutional support and resources are available. Therefore, addressing both structural challenges and individual perceptions is essential to enhance the implementation of dyslipidemia management guidelines and improve patient care outcomes.

The results of this study align with previous research indicating that patient noncompliance is a persistent barrier in managing chronic conditions such as dyslipidemia. Participants frequently reported that patients did not follow prescribed treatments or return for follow-up testing. This behavior was often accepted as a common reality of practice rather than actively addressed. From a reflective standpoint, such normalization of noncompliance may point to a deeper issue in the clinical culture where expectations for patient responsibility are low, and systematic efforts to address adherence are limited. While studies like Taniguchi et al. (2024) have documented the role of follow-up and medication adherence in treatment outcomes [24], the present findings offer contextual insight into how providers may internalize these barriers as unchangeable, which can further entrench the problem.

The current findings align with and extend prior research emphasizing the multifactorial nature of barriers to effective dyslipidemia management. As previously shown in a randomized intervention study, patients often fail to adopt dietary and physical activity changes despite targeted efforts [25], and similarly, participants in this study described persistent unhealthy lifestyle behaviors among patients. However, the present results add contextual insight by illustrating how such behaviors are often normalized within routine care. This suggests that providers may perceive nonadherence as expected rather than modifiable, potentially weakening the clinical emphasis on lifestyle counseling and leading to missed opportunities for meaningful patient engagement.

Psychological distress, such as depression and anxiety, also emerged as a critical but often overlooked factor undermining treatment adherence. Previous research has advocated for integrating behavioral health into chronic disease care as a way to reduce disparities and improve outcomes [26]. While systemic-level solutions have been proposed, the present study emphasizes the importance of embedding such integration at the provider–patient level, where lack of mental health support may directly disrupt adherence in daily clinical practice.

In addition, participants identified financial constraints as a significant barrier preventing access to medications, follow-up care, and specialized services. This reinforces evidence from earlier multi-institutional research that linked institutional and structural limitations, such as resource shortages and policy inefficiencies, to persistent gaps in guideline implementation [27]. However, our findings contribute further by illustrating how these barriers manifest specifically within the context of Jordanian hospitals, pointing to the need for locally informed policies and context-sensitive adaptations of clinical guidelines.

In addition to patient-related barriers, the findings revealed that healthcare professionals themselves face several challenges that hinder the effective implementation of dyslipidemia guidelines. Participants described a lack of proper and specialized training in dyslipidemia management, reflecting broader system-level gaps in clinical education and professional development. This observation is consistent with earlier research, which highlighted how insufficient provider training contributes to inconsistent application of evidence-based practices [27,28].

Limited familiarity with updated guidelines also appears to be a key concern. For instance, a cross-national study involving Jordanian and Egyptian educators emphasized that outdated knowledge among providers can limit the quality of care delivered [29]. In areas such as lipid management, where clinical recommendations evolve rapidly, this gap in up-to-date understanding may result in suboptimal decision-making. Furthermore, ineffective provider–patient communication was frequently mentioned as a barrier to treatment adherence. Supporting this, Tiwary and colleagues (2019) documented how breakdowns in clinical communication can lead to disengagement and negative health outcomes, particularly in chronic conditions requiring long-term management [30]. These findings suggest that strengthening provider training and improving communication practices are essential for achieving better adherence and more effective guideline implementation.

Consistent with previous findings from regional contexts, the current study identified multiple system-level barriers, including limited resources, insufficient institutional support, high workloads, and limited engagement from healthcare leadership, all of which hinder the implementation of dyslipidemia management guidelines. These findings align with those reported by Hamaideh (2017), who found that mental health nurses in Saudi Arabia encountered significant obstacles to evidence-based practice due to inadequate structural support and limited access to professional development opportunities [28]. While Hamaideh’s study focused on the mental health sector, our findings broaden this perspective by demonstrating that similar structural and institutional challenges are also prevalent in general medical practice in Jordan, particularly in the management of chronic conditions such as dyslipidemia. In the absence of sufficient infrastructure, clear institutional policies, and supportive leadership, healthcare professionals may lack both the capacity and incentive to adhere to clinical guidelines, which may contribute to inconsistencies in care delivery.

The absence of a structured follow-up system represents a significant barrier to effective dyslipidemia management because it disrupts continuity of care and reduces opportunities for timely therapeutic adjustments [31]. Additionally, the low rate of patient attendance at follow-up visits, with only 18% attending within six months after diagnosis, highlights systemic challenges related to patient engagement and healthcare coordination. These challenges undermine treatment effectiveness and limit the achievement of optimal clinical outcomes [24].

This issue is further complicated by the limited availability of essential diagnostic tools, medication shortages, and inadequate training among healthcare providers. Together, these factors contribute to inconsistent application of clinical guidelines in practice and reduce their impact on patient care [32]. Implementing integrated follow-up systems that combine technological solutions like electronic health records and automated reminders with improved resource allocation and workforce training is essential. Such measures are expected to improve patient adherence, enable early detection of treatment failures or adverse effects, and promote more consistent and effective management of dyslipidemia, leading to better health outcomes.

Heavy workload and lack of resources were consistently identified by participants as key barriers to the implementation of dyslipidemia guidelines. This finding is consistent with existing literature, which shows that healthcare providers working in high-pressure environments often do not have the time, support, or capacity to follow clinical guidelines thoroughly [33]. In such settings, immediate clinical demands tend to take priority over preventive and long-term care, which limits the attention given to chronic disease management. The current findings illustrate how time constraints, particularly among physicians, reduce opportunities for patient counseling, documentation, and critical decision-making, all of which are essential for effective guideline adherence [34]. These observations suggest that workload is not just an individual-level challenge but rather a structural issue that may require system-wide interventions. Recognizing and addressing this burden is essential for improving the practical application of evidence-based guidelines in routine care.

The lack of governmental support for healthcare institutions was identified as a critical barrier to the effective implementation of dyslipidemia guidelines. Participants described how insufficient funding, inadequate infrastructure, and limited political commitment directly affected the ability of institutions to secure essential resources, such as medications, diagnostic tools, and trained personnel. This aligns with previous research showing that health systems with weak institutional support often struggle to operationalize clinical recommendations in daily practice [32,33]. A scoping review emphasized that without adequate financial and structural backing, institutions are unlikely to sustain the processes required for guideline adherence, particularly in under-resourced settings [34]. Similarly, studies in other clinical contexts, including rare and acute care conditions, have also highlighted the role of system-level support as a key determinant of successful implementation [34,35]. The current findings reinforce the need for a supportive policy environment that ensures health institutions are equipped to integrate evidence-based guidelines into routine care.

Based on participants’ perspectives, several actionable strategies were identified to enhance the implementation of dyslipidemia management guidelines in Jordan. A major recommendation was the need for increased financial support and equitable distribution of resources. This aligns with previous research indicating that limited funding and infrastructural constraints, particularly in under-resourced systems, restrict the capacity to adopt and sustain evidence-based practices [36]. The establishment of specialized clinics and training programs targeting dyslipidemia management was also emphasized. This approach has been associated with improved cholesterol control and better adherence to clinical guidelines in similar healthcare settings [37]. Additionally, participants highlighted the importance of developing national guidelines that are adapted to the Jordanian context. Simplified content, better accessibility, and cultural relevance were viewed as essential components. These considerations are consistent with findings that stress the impact of guideline clarity, design, and contextual adaptation on successful implementation [33,34].

Enhancing patient engagement and increasing awareness were also viewed as crucial for improving guideline adherence. Suggestions included providing free lipid profile testing to support early detection and encouraging greater participation in follow-up care. Public health campaigns were viewed as essential tools to raise awareness of dyslipidemia and the importance of adhering to treatment guidelines. These insights align with evidence indicating that public education and clinician confidence are important facilitators of implementation [38,39]. In addition, a structured follow-up system was considered vital to ensure continuity of care, reinforce patient adherence, and support long-term monitoring. This corresponds with previous research highlighting the role of leadership, coordination, and organizational support in sustaining effective implementation [40]. Finally, ongoing professional development was frequently mentioned as necessary to ensure healthcare providers stay current with evolving clinical recommendations, which is consistent with reviews identifying training as a core facilitator of guideline use [38].

One of the most critical findings of this study is the perceived limited implementation of dyslipidemia management guidelines across healthcare institutions in Jordan. Although participants demonstrated awareness of these guidelines, most reported that they do not actively use them in clinical decision-making. In the absence of clear institutional direction, many professionals rely on their individual clinical experience rather than standardized protocols. This highlights a persistent gap between knowledge and practice, reflecting a broader challenge in translating evidence-based recommendations into routine care.

Such inconsistencies in guideline implementation have been previously documented and are often attributed to institutional, professional, and system-level barriers [32]. In the current study, limited implementation appeared to result from a combination of insufficient training, lack of coordination, and weak follow-up systems. Additionally, the findings revealed that nurses are often excluded from the decision-making process related to guideline use, which further limits interdisciplinary collaboration and consistent implementation. These dynamics suggest that awareness alone is not sufficient to drive practice change; rather, guideline implementation depends on system-wide support mechanisms that address both structural limitations and the distribution of clinical responsibilities within healthcare teams.

This study presents several strengths. It is among the first qualitative investigations in Jordan to examine how physicians and nurses implement the ACC/AHA and European guidelines for dyslipidemia management. By focusing on the practical implementation of these international guidelines within a local healthcare context, the study addresses a critical gap in the literature. The insights generated are especially relevant for informing the development of national, context-specific dyslipidemia guidelines that account for local barriers, resources, and clinical dynamics. Furthermore, by identifying key barriers and facilitators from the perspective of frontline healthcare providers, the study offers a foundational evidence base to support future interventions aimed at enhancing clinical implementation and improving patient outcomes.

While this study provides valuable insights into the implementation of dyslipidemia management guidelines, several limitations must be acknowledged. First, the sample size was relatively small; however, it was sufficient to achieve data saturation, which is a recognized standard in qualitative research. Second, the study was conducted in only three hospitals. Nonetheless, these included both governmental and educational institutions from two regions in Jordan, ensuring diversity in clinical settings and enhancing transferability. Third, while some clinical data, such as BMI and blood pressure, were briefly reviewed from patient medical records to provide general background context, these data were not analyzed or used in the thematic findings of the study. Fourth, the study focused on physicians and nurses, excluding other stakeholders such as pharmacists, dietitians, and patients. This was aligned with the study aim, which was to explore the perceptions and practices of direct care providers responsible for implementing the guidelines. Finally, although the interview guide was newly developed, it was informed by existing literature and expert input. Trustworthiness of the study was ensured through the use of member checking, audit trail documentation, and peer review of the analysis process. These limitations are typical in exploratory qualitative research and do not compromise the validity or credibility of the findings. Rather, they highlight directions for future, broader-scale investigations.

The findings of this study have significant implications for both policy and practice. To enhance the implementation of dyslipidemia management guidelines, healthcare policymakers in Jordan should prioritize training healthcare professionals, particularly in updated guideline content and effective patient communication. In parallel, healthcare institutions must be equipped with the necessary resources to support implementation. Investment in specialized clinics, public awareness campaigns, and the establishment of a national follow-up system are also essential to address existing barriers. Furthermore, the development of Jordan-specific guidelines that reflect local cultural and economic contexts would enable a more tailored and effective approach to dyslipidemia management. By addressing these barriers and adopting the proposed recommendations, there is considerable potential to improve the quality of care for patients with dyslipidemia in Jordan and comparable settings.

## Conclusion

The results of the current study showed a low level of implementation of any dyslipidemia management guidelines among Jordanian nurses and physicians in their clinical practices. The nurses and physicians responded difficulties they face in implementation. Besides, they gave suggestions that may help them implement dyslipidemia management guidelines. Hence, future research should focus on the development and evaluation of context-specific guidelines, the impact of specialized clinics, the use of digital tools for follow-up, and the strengthening of healthcare infrastructure. Investigating cultural, socioeconomic, and behavioral factors that influence patient adherence, as well as examining effective public health campaigns, will also be critical in advancing dyslipidemia management in Jordan and other similar settings.

## Supporting information

S1 File Open-ended questions for the focus group. Focus group semi-structured interview questions for physicians and nurses.
(DOCX)

S2 File Focus group consent form. (DOCX)

S3 File Interview experts. (DOCX)

S4 File Coding summary. (XLSX)

S1 Table Themes and sub-themes emerging form the interview data. (DOCX)
