# Supplementary material for: Exploring the implemented guidelines for dyslipidemia treatment and care among nurses and physicians: A qualitative study in Jordan
Source: PLoS One. 2025 Aug 7;20(8):e0319126. doi: 10.1371/journal.pone.0319126 (PMC12331100; doi:10.1371/journal.pone.0319126)
Supplement: S1 File — Focus group semi-structured interview questions for physicians and nurses. (DOCX) [file pone.0319126.s001.docx]

**S1. File. Open-ended questions for the focus group**

**Focus group semi-structured interview questions**

**For physicians and nurses**

1. Can you please tell me about the current updated guidelines for the Management of dyslipidemia?
2. What clinical guidelines do you currently use for dyslipidemia management for in? And why?
3. In your opinion; what is the level of your implementing specific guidelines that you follow for treating hospitalized and un-hospitalized dyslipidemia patients?
4. Can you please share with me your experience in participating in any training course on dyslipidemia guidelines?
5. In your opinion; What are the benefits of implementing a specific guideline for the management of dyslipidemia?
6. How can updated guidelines help you to manage dyslipidemia for inpatients and out patients?
7. What are the facilitators for the implementation of a specific dyslipidemia guideline in your health care setting?
8. In your opinion, what are the obstacles that are limiting the implementing of a dyslipidemia guideline in your clinical practice?
9. What are your recommendations and suggestions for implementation a specific guideline for management of dyslipidemia for inpatients and outpatients?

10. Is there anything else you would like to add?
