# Supplementary material for: Exploring the implemented guidelines for dyslipidemia treatment and care among nurses and physicians: A qualitative study in Jordan
Source: PLoS One. 2025 Aug 7;20(8):e0319126. doi: 10.1371/journal.pone.0319126 (PMC12331100; doi:10.1371/journal.pone.0319126)
Supplement: S2 File — (DOCX) [file pone.0319126.s002.docx]

**S2. File. Focus group consent form**

**Focus Group Consent Form**

You've been requested to take part in a research project. However, before you accept, you must be fully informed about the study's goal, procedures, and any benefits, dangers, or discomfort you could experience as a result of your participation.

This form contains information about the study so that you may make an informed decision about whether or not to participate.

**Participation:**

Participation in this study is completely voluntary. You are free to refuse to participate in the study or exit the study at any moment. You have the right to refuse to answer any inquiry that you do not want to answer for whatever reason.

**Study Title:**

Exploring the Implemented Guidelines for Dyslipidemia Treatment and Care among Physicians and Nurses in Jordan.

**Purpose of the Study:**

to explore the implementation of Jordanian physicians and nurses to the updated American College of Cardiology/American Heart Association (ACC/AHA) guidelines and European guidelines in the treatment and care of dyslipidemia in Jordan.

**Study Procedure:**

You will be assigned to a group of 5–10 people as part of this study. While facilitating the discussion, a moderator will ask you numerous questions. It will take 20-30 minutes. This focus group will be audio-recorded and a note-taker will be present, as approved by Al -Albayt University's Institutional Review Board.

You can choose whether or not to participate in the focus group, and you may stop at any time during the course of the study.

**Possible risks and discomforts**

No foreseeable risks are involved in this study.

**Possible study benefits**

This study is not expected to be of any direct benefits to you, but we hope to encourage more about dyslipidemia management guidelines implementations.

**Alternatives to participation**

If you do not want to be in the study, there are no alternative choices except not to take part in the study.

**Cost of participation**

There are no costs to you related to partaking in this study.

**Confidentiality of information**

We will keep confidential all research records that identify you to the extent allowed by law .no one will be identify you or your answers, and no one will know if you participate in this study. However, by signing this form you allow the research investigators to make your records available to the Institutional Review Board (IRB) Offices at Al-Albayt university as required by law.

**Contact information**

If you have any questions or concerns regarding this study, please contact

Al- Albayt University Institutional Review Board

[2021002007@st.aabu.edu.jo](mailto:2021002007@st.aabu.edu.jo)

**Documentation of Informed Consent**

I have read above information. I have asked and received answers. I agree to participate in this study.

Participant signature (or legal representative) ______________________________

Date ______________________________
