# Supplementary material for: Exploring the implemented guidelines for dyslipidemia treatment and care among nurses and physicians: A qualitative study in Jordan
Source: PLoS One. 2025 Aug 7;20(8):e0319126. doi: 10.1371/journal.pone.0319126 (PMC12331100; doi:10.1371/journal.pone.0319126)
Supplement: S3 File — (DOCX) [file pone.0319126.s003.docx]

**S3. File. Interview experts**

Participant 1 (Cardiologist): “One of the most significant barriers that affect dyslipidemia management implementation in Jordan is the lack of compliance...”

Participant 2 (Registered Nurse): “Even if I give advice to my patients, a large number of them do not follow it...”

Participant 3 (Internal Medicine Physician): “There is no good communication between patients with physicians and nurses.”

Participant 4 (Registered Nurse): “Some medications are not available or have high cost, which prevents us from implementing the guidelines.”

Participant 5 (Cardiologist): “One of the solutions is to establish a clinical center to treat hyperlipidemia like in KHCC. These patients should be referred to specialized centers.”

Participant 6 (Registered Nurse): “We don’t have enough time to implement guidelines due to workload and patient culture.”

Participant 7 (Internal Medicine Physician): “I don’t follow a specific guideline. I manage patients based on my experience.”

Participant 8 (Registered Nurse): “We have no system to follow up with patients. Once they leave the hospital, we lose contact.”

Participant 9 (Cardiologist): “Training on new guidelines is rarely conducted. We need workshops or continuing education.”

Participant 10 (Nurse): “Our role in dyslipidemia care is not clear. We just follow what doctors tell us.”

Participant 11 (Internal Medicine Physician): “Many patients cannot afford their medications, even when prescribed.”

Participant 12 (Nurse): “Patients don’t trust generic medications and prefer expensive brands.”

Participant 13 (Cardiologist): “Cultural beliefs influence patients’ attitudes towards long-term medication.”

Participant 14 (Nurse): “We don’t have enough lipid profile test kits available in our unit.”

Participant 15 (Physician): “The workload is so heavy that we barely have time for lifestyle counseling.”

Participant 16 (Nurse): “Public awareness is very low; people don't understand the importance of controlling cholesterol.”

Participant 17 (Cardiologist): “A national guideline tailored to our local system is badly needed.”

Participant 18 (Nurse): “We suggested community education campaigns to improve awareness.”

Participant 19 (Physician): “Without a structured follow-up program, we cannot track adherence.”

Participant 20 (Nurse): “Patients believe once they feel better, they can stop medication.”
