# Supplementary material for: Exploring the implemented guidelines for dyslipidemia treatment and care among nurses and physicians: A qualitative study in Jordan
Source: PLoS One. 2025 Aug 7;20(8):e0319126. doi: 10.1371/journal.pone.0319126 (PMC12331100; doi:10.1371/journal.pone.0319126)
Supplement: S1 Table — (DOCX) [file pone.0319126.s005.docx]

**S1.Table 1. Themes and sub-themes emerging from the interview data.**

| **Themes** | **Sub-themes** | **Description / Expanded Points** |
| --- | --- | --- |
| 1. Perceived different barriers that affect the implementation of dyslipidemia management guidelines in Jordan | A. Patient-related barriers | - Non-compliance with medications and lifestyle advice - Lack of follow-up and lipid profile monitoring - Psychological resistance and cultural misconceptions |
|  | B. Healthcare professional-related barriers | - Lack of training or updates on guidelines - Poor communication with patients - Limited collaboration between physicians and nurses |
|  | C. Healthcare system-related barriers | - Shortage of medical staff - Limited availability or high cost of medications - Lack of institutional support - Heavy workload and time constraints |
| 2. Perceived different suggestions and recommendations that affect implementation | A. Structural recommendations | - Establishment of specialized dyslipidemia clinics - Development of national, Jordan-specific guidelines |
|  | B. Awareness and screening | - Public awareness campaigns - Offering free lipid profile screening to the public |
|  | C. Clinical practice improvements | - Regular training programs for healthcare professionals - Organized follow-up systems for patient monitoring |
| 3. Perceived limited implementation of dyslipidemia management guidelines in Jordan | A. Implemented practice strategies | - Relying on clinical experience over standardized protocols - Use of general practices without reference to guidelines |
|  | B. Inconsistency in following guidelines | - Limited awareness of international guidelines - Absence of formal institutional policies - Partial or selective application of guideline components |
